# Uncommon Presentation of a Benign Nasopharyngeal Mass in an Adolescent: Comprehensive Review of Pediatric Nasopharyngeal Masses

**DOI:** 10.1155/2013/816409

**Published:** 2013-07-11

**Authors:** Victor M. Duarte, Yuan F. Liu, Nina L. Shapiro

**Affiliations:** ^1^Department of Head and Neck Surgery, David Geffen School of Medicine at UCLA, 62-132 Center for Health Sciences, Los Angeles, CA 90095-1624, USA; ^2^David Geffen School of Medicine at UCLA, 10833 Le Conte Avenue, Los Angeles, CA 90095, USA

## Abstract

Nasopharyngeal masses in the pediatric population are quite rare, and the majority of these are benign. In adolescent boys, there should be a high index of suspicion for juvenile nasopharyngeal angiofibromas. When malignant, the most common lesions encountered are rhabdomyosarcomas, carcinomas, and lymphomas. We report a single case from a tertiary care institution of an adolescent male with an unusual presentation of a benign nasopharyngeal mass and provide a comprehensive review of pediatric nasopharyngeal masses. Whenever possible, radiographic imaging should be obtained, in addition to biopsy, to assist in the diagnosis of pediatric nasopharyngeal masses.

## 1. Introduction

Nasopharyngeal masses in the pediatric population are quite rare, and the majority of these are benign. Differential diagnoses of nasopharyngeal masses in the pediatric population include inflammatory lesions, malignant tumors, and congenital masses. The rarity of pediatric nasopharyngeal masses and the diversity of possible pathologies make a clinical diagnosis difficult.

An understanding of the anatomy and histology of the nasopharynx is critical in deciphering the etiology of a lesion arising in this region. The nasopharynx is part of  the pharynx, which includes the oropharynx and hypopharynx, and is a part of the upper respiratory tract. Anteriorly it originates at the posterior aspect of the nasal turbinates and is perforated by the posterior nares, forming the choanae. Posteriorly and superiorly it is bordered by the skull base. Inferiorly it extends to the level of the soft palate, where the oropharynx begins. The lateral wall of the nasopharynx contains the ostium of the eustachian tube, which is surrounded by mucosa. The ostium of the eustachian tube is anterior to a pharyngeal recess known as the fossa of Rosenmuller.

The nasopharynx is lined by stratified squamous epithelium at the anterior, posterior, and inferior walls and by respiratory epithelium at the roof and nasal choanae. The remaining areas have mixtures of squamous and respiratory or intermediate (transitional) epithelium. There is abundant lymphoid tissue present, particularly at the rim of eustachian tube opening, which is functionally equivalent to that of gastrointestinal tract or mucosal-associated lymphoid tissue [1].

We present a case of an adolescent male with a nasopharyngeal mass that presented with physical and radiographic findings concerning a malignancy. Although the pathology in this was benign, this prompted a comprehensive review of pediatric nasopharyngeal masses. Given the anatomical complexity of the region and close proximity to vital structures, a biopsy of the lesion was paramount in ruling out malignancy, as clinical and radiological data would not have sufficed.

## 2. Case Presentation

A 15-year-old male was seen in the head and neck clinic complaining of progressively worsening left-sided nasal airway obstruction for several months. He reported intermittent nonfocal headaches and respiratory infections for the last 3 months. He denied otalgia, epistaxis, epiphora, pain, numbness, purulence, swelling, and anosmia. His past history was significant only for reactive airway disease.

**Figure 1:**
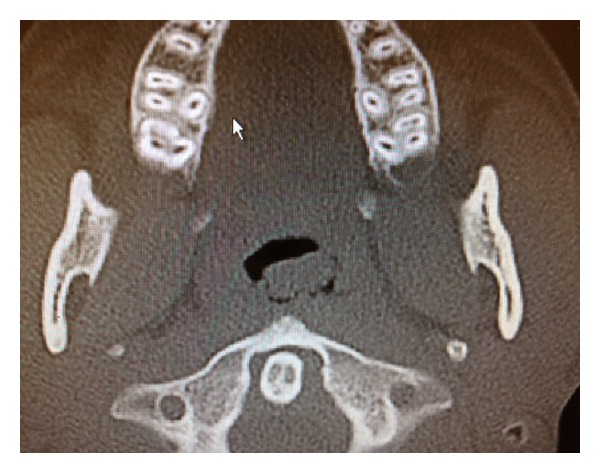
CT axial image demonstrating extension of soft tissue from the nasopharynx on the left side.

**Figure 2:**
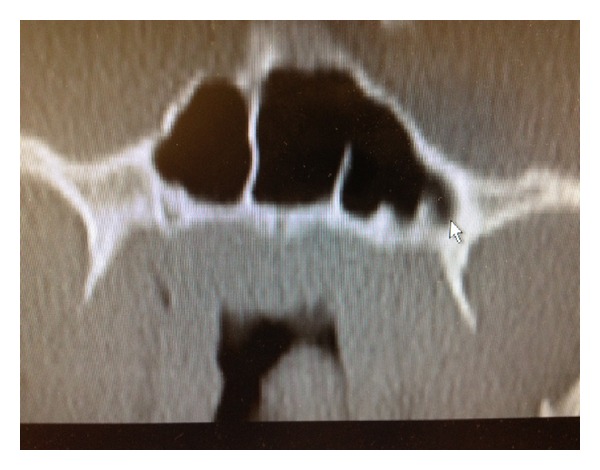
CT coronal image demonstrating a nasopharyngeal mass.

**Figure 3:**
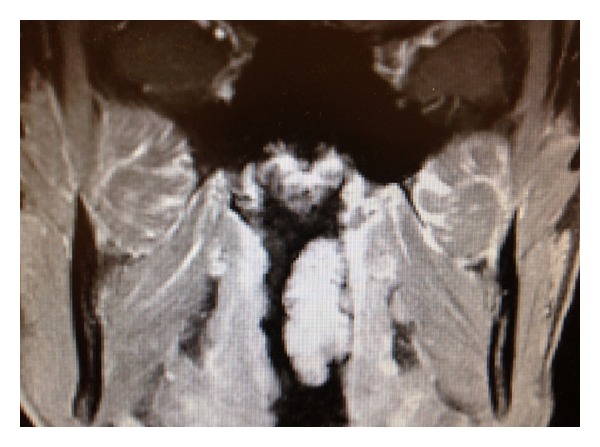
MRI coronal image demonstrating a pedunculated 3.1 × 1.7 × 1.2 cm nonvascular mass emanating from the left nasopharynx.

On physical examination, an approximately a 2 × 4 cm mass was seen posterior to the left tonsillar fossa. Nasal endoscopy demonstrated a left-sided soft tissue mass emanating from the nasopharynx. A computed tomography (CT) scan of the sinuses demonstrated some extension of soft tissue from the nasopharynx on the left side (Figures 1 and 2). Magnetic resonant imaging (MRI) demonstrated a unilateral pedunculated 3.1 × 1.7 × 1.2 cm nonvascular mass, emanating from the left nasopharynx (Figure 3).

**Figure 4:**
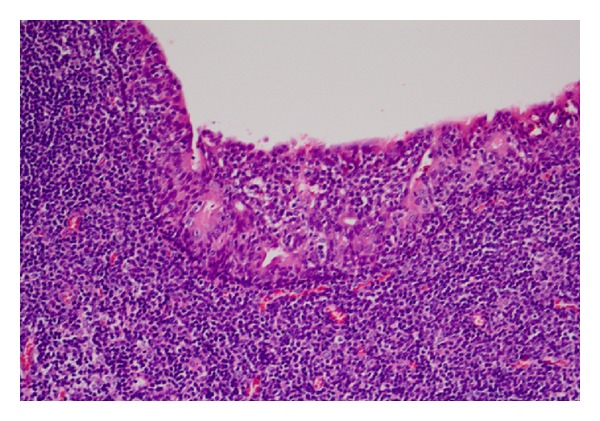
Low Power Field. Squamous mucosa with infiltrating lymphocytes.

**Figure 5:**
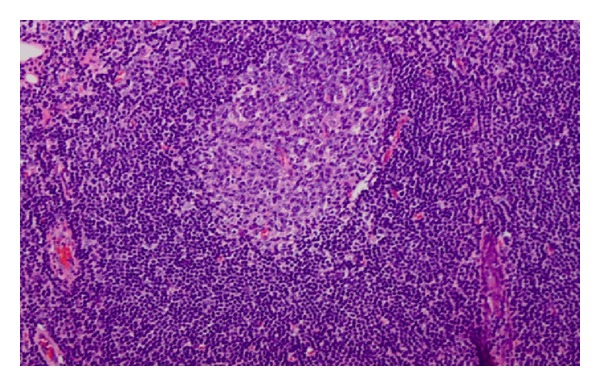
High Power Field. Reactive germinal center. Note the “polarization” of the follicle. The dark zone (at the top in this picture) is made up of immature lymphocytes (ie, centroblasts), and the light zone (at the bottom in this picture) is made up of more mature centrocytes which mature and then move out into the mantle and marginal zones further to the bottom.

The patient was brought to the operating room to evaluate his nasal cavities endoscopically. The mass was then excised via an intraoral approach for diagnostic and therapeutic purposes, given the suspicion of a malignant process, without complication. Histology demonstrated benign lymphoid hyperplasia with no evidence of malignancy (Figures 4 and 5). At 1 year followup, the patient remained asymptomatic with no evidence of disease.

## 3. Discussion

The differential diagnosis of pediatric nasopharyngeal masses includes a broad range of benign and malignant tumors, in addition to congenital lesions. Patient history and physical examination are part of the initial assessment and are the first steps in patient evaluation.

Benign masses are the most common abnormalities seen in the pediatric nasopharynx. The most common include adenoidal tissue, antrochoanal polyp (ACP), and juvenile nasopharyngeal angiofibroma (JNA). Benign adenoidal hypertrophy is by far the most common mass in the nasopharynx in children [2]. Adenoidal tissue consists of lymphoid tissue that can enlarge over time and lead to symptoms of nasal airway obstruction. This was the diagnosis in our patient. However, the unilateral nature of the mass made the case worrisome and prompted a further investigation to rule out a malignant process.

ACP is a benign lesion that arises from the mucosa of the maxillary sinus [3]. It grows into the maxillary sinus and may pass into the choanae and nasopharynx. One of the most common associated complaints is nasal obstruction. ACPs are usually unilateral and appear in childhood [3]. They account for 4–6% of nasal polyps, and 90% are solitary [3]. Microscopically, ACPs have a cystic intramaxillary portion and a solid intranasal portion. They present with less edema and possess fewer glands than inflammatory polyps [3]. They are often compared to nasal polyps, which in contrast tend to be bilateral [4]. Nasal polyps are polypoidal masses that arise from the mucous membranes of the nose and paranasal sinuses, which leads to overgrowth of the mucosa [4]. Cystic fibrosis must be ruled out in children if polyps are encountered on examination. Nasal polyps often recur due to persistence of causative factors, such as allergic rhinitis [4]. In comparison to ACPs, nasal polyposis is more commonly seen in adults [3].

JNA is the most common benign tumor of the nasopharynx and accounts for 0.05% of all neoplasms of the head and neck [5]. It is a slow growing, vascular neoplasm that occurs in adolescent males [6]. The exact site of origin of JNA remains controversial [7]. The blood supply to these benign tumors is most commonly from the internal maxillary artery, but they may also be supplied by the external carotid artery, internal carotid artery, common carotid artery, or ascending pharyngeal artery [8]. JNAs classically present with unilateral nasal obstruction, epistaxis, and nasopharyngeal mass in adolescent males, with an average age of onset of 15 years [9]. The Holman-Miller sign (also called antral sign), described as anterior bowing of the posterior wall of the maxillary antrum which is seen on cross-sectional imaging, is a finding noted on CT [10]. Angiography is critical in the evaluation of feeding vessels and allows for embolization of  JNAs [9]. Treatment options for JNAs include surgery, radiation therapy, chemotherapy, and hormone therapy, though embolization followed by surgery is still the gold standard of treatment [8].

Less commonly seen benign nasopharyngeal masses in the pediatric population include fibromas, chordomas, rhabdomyomas, and chondromas. A fibroma is a localized pedunculated mass, usually less than 1 cm in size [11]. Patients are usually asymptomatic, and excision is curative [11]. Microscopically, this mature fibrous lesion displays no evidence of infiltration of its surrounding tissue [11]. Chordomas are believed to arise from the notochord during development [12]. Histologically, chordomas display large vacuolated cells with small nuclei within a homogenous intercellular substance (physaliphorous cells), with rare mitoses [12]. Chordomas have been reported to recur as high grade spindled cell sarcomas and stain positive for cytokeratin and EMA [12]. Rhabdomyomas are benign neoplasms of striated muscle, which are divided into fetal and adult types based on histologic features [13]. Histologically, the fetal type is immature, with slender muscle fibers and primitive spindle shaped mesenchymal cells, with no evidence of pleomorphism or mitotic figures [13]. The adult type displays clear cytoplasm (glycogen), finely granular cytoplasm, and sparse striations and is rarely seen in the nasopharynx [14]. A chondroma is a benign cartilaginous tumor that occurs in two forms, solitary and multiple, and is difficult to differentiate from a malignant chondrosarcoma [15].

Congenital midline nasal masses include nasal dermoids, nasal gliomas, and encephaloceles. These congenital anomalies are estimated to occur in 1 out of  20,000–40,000 births [16]. Although rare, these lesions are clinically important because of their potential for communicating with the central nervous system [16]. When a patient presents with a mass consistent with any of these three lesions, biopsy of the mass should not be performed prior to obtaining imaging [16]. A dermoid cyst is a midline lesion that can present as a mass on the dorsum of the nose or solely intranasally [16]. They often have a pit or sinus tract opening on the nasal dorsum, hair around the external opening, and discharge of sebaceous material or purulence [16]. It has been known to extend intracranially and cause central nervous system infections, such as meningitis [16].

Nasal gliomas are firm masses which are nonpulsatile and tend to arise from the lateral nasal wall [16]. An important distinguishing feature of these lesions is their lack of enlargement with bilateral compression of the internal jugular veins [16]. In contrast, encephaloceles are blue colored, often pulsatile lesions that arise from the nasal bridge and can lead to nasal broadening. They are compressible and can be transilluminated on exam [16]. Furthermore, they can enlarge with crying or with bilateral compression of the internal jugular veins. Intranasal encephaloceles can be seen arising from the cribriform plate [16]. Microscopically, they have a cystic intramaxillary portion and a solid intranasal portion [16].

Other less commonly seen congenital lesions that have been reported in the pediatric nasopharynx include teratomas, craniopharyngiomas, Thornwaldt's cysts, hamartomas, and hemangiomas. Teratomas, also known as hairy polyp of nasopharynx, are derived from pluripotent tissues composed of all 3 germinal layers and are often diagnosed in the neonatal or infancy periods [17]. The lesion can be sessile or pedunculated and often can be seen protruding into the mouth [17]. Teratomas have been associated with elevated serum alpha fetoprotein levels along with intracranial anomalies including palatal fissures, hemicranias, anencephaly, and polyhydramnios [17]. On CT or MRI, teratomas may demonstrate cystic areas as well as solid areas with bone and tooth formation [18].

A craniopharyngioma is a type of pituitary adenoma which is cystic in nature. The cyst contains brownish fluid with refractile cholesterol crystals [19]. It is a benign epithelial tumor of the central nervous system that is found as a sellar or suprasellar mass. It has been reported to recur after excision, although it rarely undergoes malignant change [20]. Thornwaldt's cyst is a relatively rare lesion located in the posterior wall of the nasopharynx [21]. Most are small and asymptomatic, but some can cause nasal obstruction, postnasal drip, occipital headache, or eustachian tube dysfunction. Treatment is surgical if the patient remains symptomatic. A hamartoma is a simple congenital malformation derived from local tissue and often appears polypoid but does not infiltrate the surrounding tissue [22]. Nasopharyngeal hemangiomas have also been reported in the literature but are exceedingly rare [23].

Malignancies of the pediatric nasopharynx include rhabdomyosarcomas, nasopharyngeal carcinomas (NPCs), and lymphomas. Malignant tumors of the nasopharynx are rare in this age group, and the histology generally varies with the age of the patient at presentation. Soft tissue sarcomas and lymphomas are more commonly diagnosed in younger children, whereas carcinoma of the nasopharynx has a predilection for adolescents and teenagers [24]. Rhabdomyosarcomas are most commonly seen in the nasopharynx, orbit, middle ear/mastoid, nose, or paranasal sinuses [25]. Seventy-five percent of patients with rhabdomyosarcomas are diagnosed at age 12 or younger. These tumors are rarely found in teenagers, adults, or the elderly [26]. Eighty-five percent of rhabdomyosarcomas are of the embryonal subtype, including botyroides variant [27]. Survival varies by site: orbit—90%; nose, paranasal sinuses, and nasopharynx—45%; other head and neck subsites—75% [28]. Histologically, it is described as a highly cellular spindle cell tumor with frequent mitotic activity, staining positive for desmin, myosin, myoglobin, myogenin, and myoD1 markers [28].

NPC accounts for 5% of all pediatric head and neck malignancies and for about one-third of all cancers of the upper airway [29]. It is very uncommon in children younger than 10 years of age but increases in incidence from 0.8 to 1.3 per million per year in children of ages 10 to 14 and in children of ages 15 to 19, respectively [30]. The incidence of NPC is characterized by racial and geographic variations, with an endemic distribution among well-defined ethnic groups, such as inhabitants of some areas in North Africa and Southeast Asia. In the United States, NPC is overrepresented in black children when compared with other malignancies [31]. NPC is strongly associated with Epstein-Barr virus (EBV) infection. Three histologic subtypes of nasopharyngeal carcinoma are recognized by the World Health Organization (WHO): type 1 is squamous cell carcinoma; type 2 is nonkeratinizing squamous cell carcinoma; and type 3 is undifferentiated carcinoma [24]. Children with NPCs are more likely to have WHO type 2 or type 3 tumors [24]. The overall survival of children and adolescents with NPC has improved over the last four decades with 5-year survival rates in excess of 80% [31, 32]. Non-Hodgkin's lymphoma (NHL) accounts for 60% of all lymphoma cases [33]. It is associated with T-cell deficiencies [34]. They are often bulky lesions that affect multiple sinuses and nasal cavity, with extension into nasopharynx. The 5-year survival is 55% for stage I/II [35]. The different histological subtypes include NK/T cell, diffuse large B cell, and peripheral T cell; mantle cell lymphoma is most common in the nasopharynx.

Management of benign tumors is usually surgical, but the approach may vary dramatically depending on the histology and the anatomical pattern of involvement. Malignant tumors are generally treated with radiation therapy and/or chemotherapy. In summary, the necessity of biopsy in the evaluation of pediatric nasopharyngeal masses should be evident given the large spectrum of potential diagnoses in the pediatric nasopharynx. It is the histology which will most influence the physician's choice of therapy.

## 4. Conclusion

Although the majority of nasopharyngeal masses in the pediatric population are benign, they must be fully evaluated in order to rule out malignancy based on clinical and radiologic findings. A mass in this region can be safely excised only after careful preoperative evaluation.
